# Adapting Peer Researcher Facilitated Strategies to Recruit People Receiving Mental Health Services to a Tobacco Treatment Trial

**DOI:** 10.3389/fpsyt.2022.869169

**Published:** 2022-05-26

**Authors:** Amanda L. Baker, Kristen McCarter, Lisa Brophy, David Castle, Peter J. Kelly, Nadine Cocks, Melissa L. McKinlay, Catherine Brasier, Ron Borland, Billie Bonevski, Catherine Segan, Donita E. Baird, Alyna Turner, Jill M. Williams, Erin Forbes, Laura Hayes, John Attia, David Lambkin, Daniel Barker, Rohan Sweeney

**Affiliations:** ^1^School of Medicine and Public Health, College of Health, Medicine and Wellbeing, University of Newcastle, Callaghan, NSW, Australia; ^2^School of Psychological Sciences, College of Engineering, Science and Environment, University of Newcastle, Callaghan, NSW, Australia; ^3^Social Work and Social Policy, School of Allied Health, Human Services and Sport, La Trobe University Melbourne, Melbourne, VIC, Australia; ^4^Centre for Mental Health, Melbourne School of Population and Global Health, The University of Melbourne, Melbourne, VIC, Australia; ^5^Centre for Complex Interventions, Centre for Addiction and Mental Health, Department of Psychiatry, University of Toronto, Toronto, ON, Canada; ^6^Illawarra Health and Medical Research Institute and the School of Psychology, University of Wollongong, Wollongong, NSW, Australia; ^7^Research, Advocacy and Policy Development, Mind Australia Limited, Heidelberg, VIC, Australia; ^8^Department of Mental Health, St Vincent's Hospital Melbourne, Fitzroy, VIC, Australia; ^9^Melbourne School of Psychological Sciences, The University of Melbourne, Melbourne, VIC, Australia; ^10^Cancer Council Victoria, Melbourne, VIC, Australia; ^11^Flinders Health and Medical Research Institute (FHMRI), College of Medicine & Public Health, Flinders University, Bedford Park, SA, Australia; ^12^Melbourne School of Population and Global Health, The University of Melbourne, Melbourne, VIC, Australia; ^13^School of Medicine, IMPACT, Institute for Innovation in Physical and Mental Health and Clinical Translation, Deakin University, Geelong, VIC, Australia; ^14^Division of Addiction Psychiatry, Rutgers Robert Wood Johnson Medical School, New Brunswick, NJ, United States; ^15^Hunter Medical Research Institute, Newcastle, NSW, Australia; ^16^Centre for Health Economics, Monash Business School, Monash University, Melbourne, VIC, Australia

**Keywords:** tobacco treatment, smoking cessation, quitline, peer worker, mental illness, recruitment, cost analysis, severe mental illness (SMI)

## Abstract

**Introduction:**

One of the most challenging aspects of conducting intervention trials among people who experience severe mental illness (SMI) and who smoke tobacco, is recruitment. In our parent “QuitLink” randomized controlled trial (RCT), slower than expected peer researcher facilitated recruitment, along with the impact of COVID-19 pandemic restrictions, necessitated an adaptive recruitment response. The objectives of the present study were to: (i) describe adaptive peer researcher facilitated recruitment strategies; (ii) explore the effectiveness of these strategies; (iii) investigate whether recruitment strategies reached different subgroups of participants; and (iv) examine the costs and resources required for implementing these strategies. Finally, we offer experience-based lessons in a Peer Researcher Commentary.

**Methods:**

People were included in the RCT if they smoked at least 10 cigarettes a day and were accessing mental health support from the project's two partnering mental health organizations in Victoria, Australia. The majority of people accessing these services will have been diagnosed with SMI. Recruitment occurred over 2 years. We began with peer facilitated recruitment strategies delivered face-to-face, then replaced this with direct mail postcards followed by telephone contact. In the final 4 months of the study, we began online recruitment, broadening it to people who smoked and were accessing support or treatment (including from general practitioners) for mental health and/or alcohol or other drug problems, anywhere in the state of Victoria. Differences between recruitment strategies on key participant variables were assessed. We calculated the average cost per enrolee of the different recruitment approaches.

**Results:**

Only 109 people were recruited from a target of 382: 29 via face-to-face (March 2019 to April 2020), 66 from postcards (May 2020 to November 2020), and 14 from online (November to December 2020 and January to March 2021) strategies. Reflecting our initial focus on recruiting from supported independent living accommodation facilities, participants recruited face-to-face were significantly more likely to be living in partially or fully supported independent living (*n* = 29, <0.001), but the samples were otherwise similar. After the initial investment in training and equipping peer researchers, the average cost of recruitment was AU$1,182 per participant—~US$850. Face-to-face recruitment was the most expensive approach and postcard recruitment the least (AU$1,648 and AU$928 per participant).

**Discussion:**

Peer researcher facilitated recruitment into a tobacco treatment trial was difficult and expensive. Widely dispersed services and COVID-19 restrictions necessitated non-face-to-face recruitment strategies, such as direct mail postcards, which improved recruitment and may be worthy of further research.

**Clinical Trial Registration:**

The trial is registered with ANZCTR (www.anzctr.org.au): ACTRN12619000244101 prior to the accrual of the first participant and updated regularly as per registry guidelines. The trial sponsor was the University of Newcastle, NSW, Australia.

## Introduction

Smoking rates are much higher among people who experience severe mental illness (SMI) compared to the general population (1). Consequently, people with SMI experience poorer quality of life related to smoking (2) and die prematurely from smoking related diseases (3). There is strong evidence that tobacco treatment consisting of cognitive behavior therapy (CBT) and pharmacotherapy (such as nicotine replacement therapy (NRT) and varenicline) are associated with reductions in smoking and cardiovascular disease risk among people with SMI (4) and that such interventions can be delivered effectively by telephone (5). However, use of quitlines tends to be low relative to their potential reach (6) and research regarding scalable, low-cost efforts to increase quitline reach is critically needed to reduce tobacco related health disparities (7).

Peer workers are individuals who provide services in mental health and/or substance use treatment settings informed by their own experience of recovery from mental illness and/or substance use and skills obtained from formal peer worker training (8, 9). We have previously reported on the successful delivery of a peer worker delivered smoking cessation and healthy lifestyle intervention among people who experience SMI (10). In that feasibility study, we received 104 referrals from one community mental health organization, with 43 people being included in the study over a 1-year period (10). Dickerson et al. (11) reported a pilot trial which recruited 30 people with SMI into a peer delivered smoking intervention over a 6-month period (11). Given the positive but modest reach of peer delivered smoking cessation interventions and the potential for higher reach of quitlines, peer workers may be able to enhance reach of quitlines to people with SMI, by identifying smokers within mental health services and facilitating referral to quitlines. As such, we conducted a randomized controlled trial (RCT, the “Quitlink Project”) evaluating the effectiveness and cost-effectiveness of a peer worker facilitated intervention for smoking among people accessing support for SMI (12, 13).

One of the most challenging aspects of conducting intervention trials among people who experience SMI is recruitment (14, 15). Challenges include: the necessity for extensive collaborations between researchers, consumers, health staff and institutions, each with their own expectations and concerns; clinicians' concerns about consumers' vulnerability and reduced decision-making ability; and consumer doubts about potential benefits in the face of lengthy research procedures (16). High caseloads and clinical staff feeling they do not have the necessary knowledge of research to feel comfortable discussing this with service users have also been identified as barriers to recruitment (17). In their systematic review of recruitment strategies in mental health trials, Liu et al. (16) found only two RCTs cited among people who experience SMI that formally assessed the effectiveness of different strategies to improve recruitment (18, 19). Neither multi-media consent procedures nor co-designed participant invitation leaflets were associated with improved recruitment. Clearly, in the face of numerous challenges, recruitment into a study needs to be appealing to staff and consumers and not represent a burden for staff.

There are additional challenges recruiting for studies of interventions for substance use, like smoking, due to high levels of ambivalence among potential participants. People experiencing SMI who smoke report being interested in quitting but may not feel ready to do so in the short-term (20). Recruitment processes need to be sufficiently attractive to people who do and do not want to quit smoking in the near future.

Globally, individuals receiving treatment for mental illness and/or substance use have faced unprecedented challenges during the coronavirus (COVID-19) pandemic. They are at elevated risk of vulnerability to COVID-19 associated with co-occurring health conditions and mental health sequelae arising from isolation and socioeconomic instability (21). During the pandemic, there was a significant divergence from routine care in Australia, with increased use of telehealth. Given our interest in potentially linking people with SMI who smoke to quitline, the characteristics of those who enrolled in our study are of interest and may help inform further uptake of the use of quitlines, post-pandemic.

Peer workers are strong role models for clients, and are particularly successful in developing hope, promoting self-esteem and empowering consumers (22). These unique skills are likely to be extremely valuable in helping to promote engagement of people with SMI within quitline services (23). In our Quitlink Project, peer workers were engaged as peer researchers, recruiting participants, collecting baseline data and delivering brief advice. We initially developed a peer delivered (face-to-face) recruitment strategy. Slower than expected recruitment and social distancing requirements and other restrictions of the COVID-19 pandemic necessitated an adaptive recruitment response, involving progressively less intensive peer facilitation. As described further below, peer facilitation was adapted from face-to-face contact with participants, to direct mail postcards with telephone contact, and then online peer researcher video presentations. Inclusion criteria were also modified to broaden the reach of the project beyond the original two mental health partner organizations involved and baseline assessment was abbreviated to allow administration via telephone and online.

There is a marked lack of evidence on the costs and recruitment effectiveness for tobacco treatment trials targeting people who experience mental illness (15, 16). Potential insights may be drawn from smoking recruitment studies amongst general populations (24) and Liu et al's. (16) systematic review of recruitment studies for mental health trials (i.e., not tobacco treatment studies). Buller et al. (24) found online advertisements could be a relatively effective and inexpensive recruitment approach (US$43.35 per enrollee, *N* = 1,426) compared with recruiting via quitline screening (US$133.61, *N* = 149). Liu et al. (16) found web-based advertisements could be an inexpensive way to recruit people to mental health RCTs compared with generating referrals from specialized care or primary care (UK£13.41 vs. UK£183.24 vs. UK£407.65 per patient enrolled, respectively). We anticipated recruiting to our study would be relatively more resource intensive than these studies, given we aimed to target people who experience both mental illness and tobacco dependence. Here we report on three different peer facilitated recruitment strategies, and the associated costs. The peer researchers employed on the project also report on their experiences of recruitment in a Peer Researcher Commentary.

## AIMS

The objectives of the present study were to: (i) describe adaptive peer researcher facilitated recruitment strategies; (ii) explore the effectiveness of recruitment strategies in terms of recruitment number and rate of accrual; (iii) investigate whether recruitment strategies reached participants with different demographic, smoking and clinical characteristics; and (iv) examine the costs and resources required for implementing these strategies. Finally, we offer experience-based lessons from peer researchers for recruiting people who experience SMI into a tobacco treatment trial.

## Methods

### Participants

As described in our protocol paper (12), to be eligible, participants smoked at least 10 cigarettes a day and were accessing treatment or support from participating mental health agencies. The majority of people accessing these services will have been diagnosed with SMI, such as schizophrenia, schizoaffective disorder, bipolar disorder, delusional disorder and depressive disorders. Exclusion criteria were: current engagement in Quitline Victoria's callback service; no ready access to a telephone; inability to complete informed consent and/or the screening survey; acute suicidality; contraindications to nicotine replacement therapy (NRT); and pregnancy. When online recruitment commenced (as described below) inclusion criteria were expanded to include people accessing support or treatment, including from their general practitioner, for a mental health and/or alcohol or other drug use condition. The target sample was 382 randomized to Quitline and NRT support or generic support.

### Partnerships With Mental Health Organizations

Recruitment began by partnering with two mental health organizations in Victoria, Australia. Two chief investigators (DC and LB) were employed or funded by these organizations at the beginning of the project and they worked with the two peer researchers to promote research participation within the services. The Ethics Committee at one of these sites was the primary Ethics Committee for the study (St Vincent's Hospital, Melbourne, HREC Reference Number: HREC/18/SVHM/154). Ethics approval was also obtained from the University of Newcastle HREC (HREC Reference Number: H-2018-0192) and the Cancer Council Victoria, HREC (HREC Reference Number: 1807).

Peer researchers were supervised by ALB (a clinical psychologist) weekly in group or individual teleconferences or videoconferences, depending on overlapping days of work. When ALB was unavailable, another clinical psychologist investigator (PJK) led supervision. The Research and Evaluation Manager (LH) at one of the participating mental health organizations attended monthly team investigator meetings and some peer research supervision sessions. As described below, each mental health organization provided current consumer names and contact details to peer researchers for recruitment.

### Recruitment Procedure

Recruitment occurred over 2 years (from March 2019 to April 2021), and strategies were adapted in response to slow face-to-face recruitment (pre-COVID 19) and to social distancing and lockdowns prohibiting face-to-face access during the COVID-19 pandemic (from March 2020). The state of Victoria experienced 11 lockdowns during the recruitment period, precluding further face-to-face recruitment.

### Recruitment Strategies

Recruitment strategies were adapted over the course of the trial as described below.

1) Face-to-face via peer researchers (March 2019 to April 2020)

The initial (pre-COVID-19) recruitment method employed peer researchers as described previously (12). Two peer researchers (NC and MMc) were each employed 2 days per week, with one increasing to 3 days per week during the last 6 months of recruitment. A third peer researcher (CB) assisted with administrative aspects of recruitment half a day a week as recruitment progressed. Peer workers assisted with a parallel qualitative study when they had spare time. Their work was guided by a peer manual co-designed by NC; it contains introductory scripts and detailed descriptions of study procedures. Both peer researchers received two and a half days face-to-face training on recruitment procedures by chief investigators (ALB and PJK) and one peer researcher received an additional 3 days training (by EF) on using an iPad for data entry etc. Peer researchers were observed visiting initial sites, conducting baseline assessments and delivering brief advice. Two peer researchers had formerly smoked and the other had never smoked.

Peer researchers visited various sites of the two partnering mental health organizations presenting information to staff and potential participants about the study and leaving postcards about the study and consent-to-contact forms. Supported independent living accommodation facilities were targeted, and some community services were also visited. Service staff were asked to refer potential participants using the consent-to-contact forms. This stage of recruitment also included advertising (e.g., flyers in residential and community services and online service newsletters). Peer researchers used iPads to guide potential participants through eligibility and consent procedures and to gather baseline data via REDCap (a secure web-based application designed to support data capture for research studies). Baseline data collection took around 1.5 h per participant.

2) Direct mail postcard (May 2020 to November 2020)

The second recruitment method involved two staggered direct mail postcard campaigns to all people registered with the project's two partnering mental health organizations (smoking status is not recorded on organization registers). Postcards were developed in conjunction with peer researchers (see Supplementary Material). The first postcard contained the project logo and brief information about the project and contact details of the peer researchers. The second postcard was the same except for a new background photo of two people in conversation. Postcards invited people registered with either participating mental health organization who smoked at least 10 cigarettes per day to telephone peer researchers to find out more about the study. Peer researchers obtained verbal consent via telephone to participate in the study and conducted an abbreviated baseline assessment. A shorter baseline assessment was necessary due to the assessment interview being conducted over the phone, to reduce participant burden, and took about 30–45 min. Both of the postcard mail outs were staggered over 3 months (May to July 2020 and September to November 2020) in order to accommodate availability of peer researchers to respond to potential participants. A postage service used by one of the mental health organizations was paid to send the postcards.

3) Online (November to December 2020 and January to March 2021)

With our recruitment rate improved by direct mail postcards (as described below) but still lower than anticipated, we broadened recruitment beyond the initial two mental health organizations. Ethics permission was granted to extend recruitment to people who smoked and were accessing support or treatment (including from general practitioners) for mental health and/or alcohol or other drug problems, anywhere in the state of Victoria. A study website was developed and the study was advertised via paid advertisements on Facebook, newsletters of community organizations and professionals and a register of substance use studies. Social media posts are attached in Supplementary Material. Online recruitment involved investigator and peer researcher videos explaining the study and consent procedures, asking interested people to either telephone peer researchers or complete online screening, consent and baseline assessments.

### Assessment Measures

After completing consent procedures, participants completed a number of measures as part of baseline assessment, with selected measures including demographic characteristics (gender, age, relationship status, employment status, and accommodation status), smoking, mental health, alcohol use and quality of life. Psychometric properties of the measures employed have been described previously (12). Measures selected for the present study are summarized below.

#### Smoking

Self-reported data regarding cigarettes smoked per day (for daily smokers) or cigarettes per week (for non-daily smokers) were collected. The two item Heaviness of Smoking Index (HSI) assessed nicotine dependence (25, 26). It uses a six-point scale calculated from the number of cigarettes smoked per day (1–10, 11–20, 21–30, 31+) and the time to first cigarette after waking (≤5, 6–30, 31–60, and 61+ minutes). Nicotine dependence is then categorized into a three-category variable: low (0–1); medium (2–4); and high (5–6).

#### Mental Health

The 10-item Kessler Psychological Distress Scale [Kessler-10; (27)] measures non-specific psychological distress. Low scores (10–15) indicate little or no psychological distress and higher scores indicate increasing levels of distress (moderate, 16–21; high, 22–29; and very high, 30–50).

The Mini International Neuropsychiatric Interview [MINI; (28)] was administered to obtain lifetime mental health diagnosis; this was administered at the 2-month follow-up to reduce assessment burden at baseline. The McLean Screening Instrument for Borderline Personality Disorder (29) was administered to four people to verify their self-reported main diagnosis of borderline personality disorder. Of these, two were negative (on the MINI and McLean), one had a psychotic disorder according to the MINI, and the remaining person screened positive for borderline personality disorder. Diagnoses were grouped into “psychotic” and “non-psychotic” disorders. Psychotic disorders were bipolar 1 disorder, bipolar 1 disorder with psychotic features, any psychotic disorder (includes schizophrenia), and major depressive disorder with psychotic features. Non-psychotic disorders were major depressive disorder, agoraphobia, obsessive-compulsive disorder, posttraumatic stress disorder, generalized anxiety disorder and borderline personality disorder.

#### Alcohol Use

The Alcohol Use Disorders Identification Test – Brief [AUDIT-C; (30)], a three item screening tool, was used to identify hazardous alcohol use or active alcohol use disorder. It is scored on a 0-12 scale with a cut off of 3 (women) or 4 (men), indicative of hazardous drinking or alcohol use disorder.

#### Quality of Life

Health related quality of life (HRQL) scores [utilities, (31)] were elicited using the 35-item Assessment of Quality of Life-8 Dimension (AQoL-8D) (32, 33) for participants who were recruited face-to-face. To address concerns about the length of the assessment, when we transitioned to recruitment via postcard and online, we transitioned to elicit HRQL utilities using the EQ-5D-5L (34) plus four AQOL-8D question bolt-ons. These can be used in combination to calculate HRQL utilities and has been shown to be comparable to the AQOL-8D (35). Utilities are anchored by 1=perfect HRQOL and 0=death.

### Costing Analysis

Following the costing principles set out in the Consolidated Health Economic Evaluation Reporting Standards (CHEERS), we describe the resources and associated costs used to recruit participants effectively up to the point of completing baseline assessment (36). Taking the perspective of the research project, we present a breakdown of costs over four phases as they occurred in the study: (i) training and equipping peer researchers; (ii) face-to-face recruitment; (iii) postcard recruitment; and (iv) online recruitment. We then calculate the average cost per participant recruited via our package of three strategies and also the average cost per participant recruited via each strategy independently. Our investment in training and equipping peer researchers will have wider use beyond this trial and are thus excluded from average cost per participant calculations—akin to research groups engaging peer researchers already trained in RCT recruitment and implementation (24). Costs include personnel time (investigators, peer researchers, administrative support staff), iPad and mobile phone costs, travel costs associated with training, supervision, and peer researcher site visits, costs of designing and printing advertising materials, postcard design and mail-out costs, website design and hosting costs plus on-line recruitment advertising costs, and the costs of giftcard vouchers given to participants after completing baseline assessment. We exclude the costs of investigator time in completing ethics amendments to adapt recruitment methods, and constructing the baseline survey, which will have future use. All costs are presented in 2021 Australian dollars (AU$). See Supplementary Material for more detail of included costs and data sources.

### Statistical Analyses

Descriptive statistics are presented as counts (%) and means (standard deviation; SD). An alpha level of 0.05 was specified for all tests and confidence intervals. The data were analyzed in SAS v9.4.

Differences in demographic, smoking and clinical characteristics of participants between recruitment methods were assessed using one-way ANOVA for continuous variables and Fisher's exact test for categorical variables. A Bonferoni correction was used to adjust for multiple comparisons, resulting in an adjusted alpha level of 0.005 for significance. Post-hoc tests were carried out on any significant results. Pairwise comparisons of categorical variables were assessed using Fisher's exact test, with effect sizes shown as odds-ratios (95% CI). Pairwise comparisons of continuous variables were assessed using Welch's t-test, with mean differences (95% CI).

## Results

**Figure 1:**
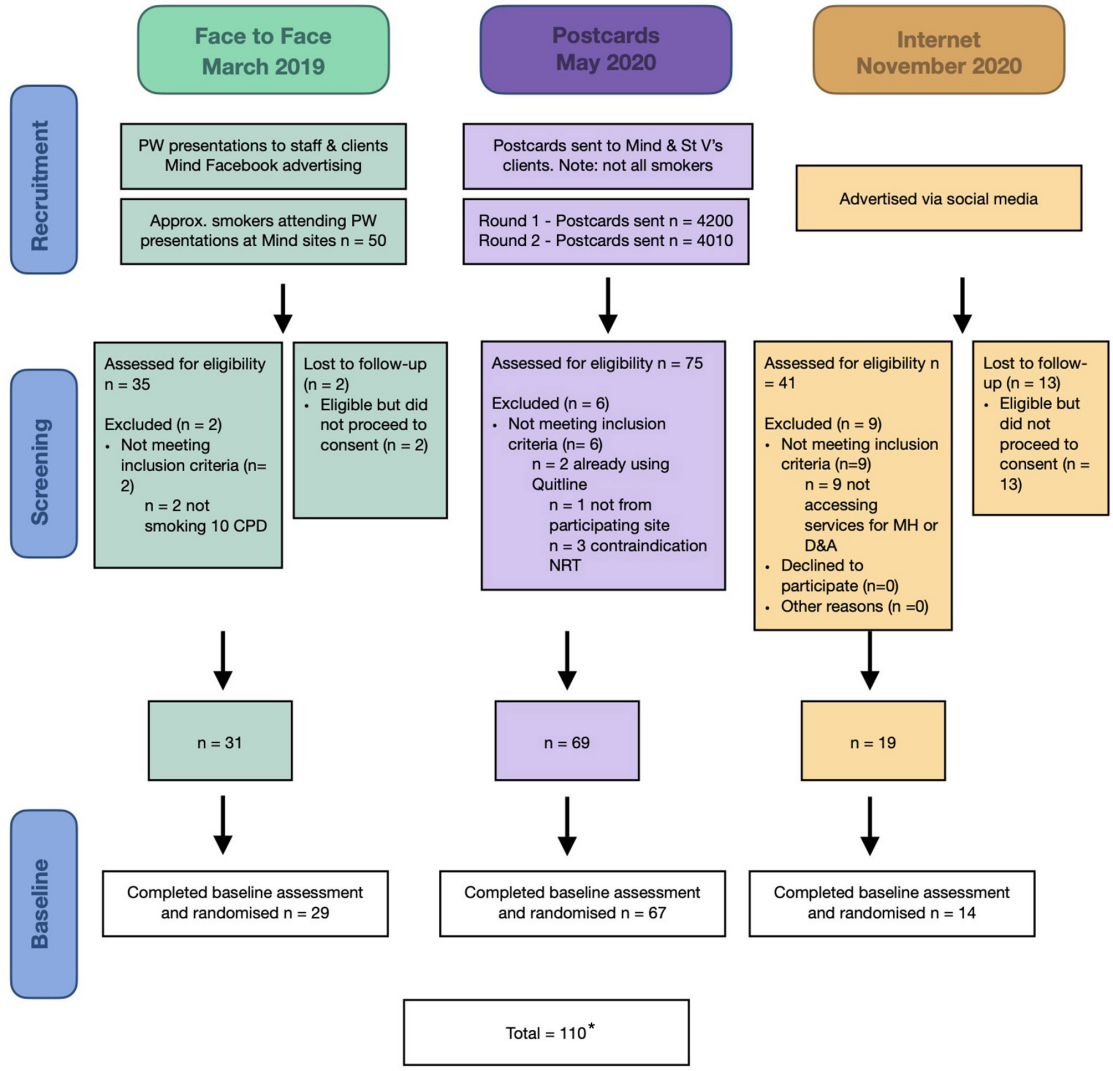
CONSORT diagram. *One person withdrew from the study.

**Figure 2:**
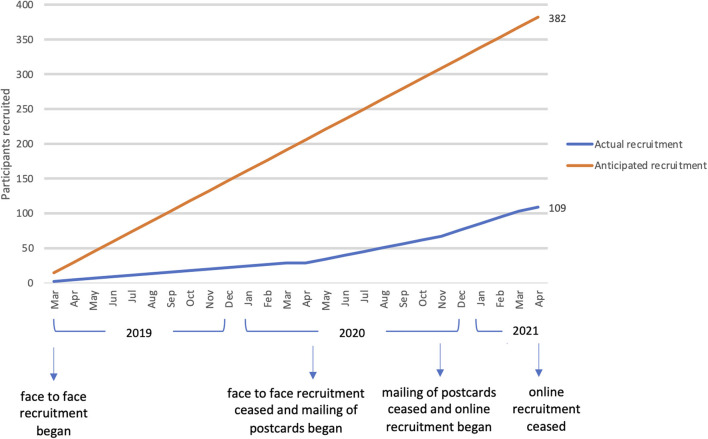
Recruitment per month according to recruitment strategy.

A total of 110 of our projected sample of 382 participants completed consent procedures and baseline assessments and were randomized. One person subsequently withdrew, leaving a total sample of 109 people. Our recruitment target had been 16 people per month. See Figure 1 for a summary of recruitment figures. Figure 2 shows recruitment per month according to recruitment strategy.

In the first, face-to-face, recruitment period between March 2019 and April 2020 (13 months) ~50 people (based on estimates by peer researchers) who smoked attended peer researcher presentations within the participating mental health organizations. Of these, 35 were assessed for eligibility. Two were excluded (not smoking 10 cigarettes per day) and two were unable to be contacted to complete consent, yielding 31 eligible people of whom 29 completed baseline assessments and were randomized. At the first mental health organization, this phase of recruitment focused on visiting six supported independent living services (from which 14 people were recruited; range 1–5), six community centers for mental health and well-being with two of these via video as they were in rural locations (none recruited), five residential rehabilitation facilities for youth (four recruited from two services; range 1–3); an accommodation support service (two recruited); and an adult outreach service (none recruited). At the second mental health organization, posters were placed in an inpatient ward (no recruits), two outpatient clinics (none recruited), and a short-stay sub-acute residential facility (three recruited). In total, 23 of 29 people recruited during this face-to-face phase were recruited from residential services; the monthly recruitment rate was 2.2.

In the direct mail postcard recruitment phase, a total of 4,200 postcards were mailed between 1 May and 30 July 2020 to all people registered with the two mental health organizations participating in the study. A second postcard was sent (minus ‘return to sender’ addresses and those who requested no further postcards after the first mail out) to 4010 people between 17 September and 5 November 2020. Interested people responded to postcards over the 6 months that direct mail occurred but were welcome to respond until April 2021, when recruitment closed (12 months). A total of 75 people were assessed for eligibility. Six people were excluded (two were already using quitline, one was not from a participating site and three reported contraindications to NRT). A further two people did not complete baseline assessment, leaving a total of 67 participants recruited via direct postcard mail out. As mentioned above, one person subsequently withdrew their data from the study, leaving 66 people recruited via postcard. Recruitment was higher in the second mailout. During the first mailout we received the following number: June 2020 (5); July 2020 (4); August 2020 (12). During the second mail out we received: September 2020 (3); October 2020 (19); November 2020 (8); December 2020 (4); January 2021 (2); February 2021 (3); March 2021 (4); April 2021 (3). The recruitment rate per month was 5.5 people per month over the 12 month response period, still lower than the target needed to fill the sample, even if used over the entire recruitment period.

Online recruitment commenced 12 November 2020, temporarily closed between 21 December 2020 and 10 January 2021 due to Christmas vacation and recommenced on 11 January 2021, continuing until 31 March 2021 (total of four months). Facebook advertisements generated a total of 476,727 impressions (defined as an advertisement appearing on a user's page), reaching 121,467 unique individuals, with 5009 link clicks. A total of 41 people were assessed online for eligibility, with nine ineligible (not currently accessing any services or support for mental health and/or AOD problems), 13 not proceeding to consent, and five not completing baseline, leaving a total of 14 recruited into the study. The recruitment rate per month was 3.5. All people recruited chose to complete the baseline assessment online (rather than request assistance from a peer researcher).

### Characteristics of the Participant Group

Demographic, smoking, mental health, and health related quality of life (HRQL) data are presented below. As seen in Table 1, the sample was evenly divided between men and women, aged in their mid-forties, most were unemployed, and most were not married or cohabiting. Not shown in Table 1, 50 (45.9%) had no further education after leaving school and 67 (61%) were receiving a disability pension. Most (*n* = 99; 91%) endorsed English as the main language spoken at home. Only three people (2.8%) identified as Aboriginal or Torres Strait Islander.

**Table 1:** Participant characteristics by recruitment strategies.

**Variable**	**Face-to-face**	**Postcard**	**Online**	**Total**	***p***
	**(*****n*** **=** **29)**	**(*****n*** **=** **66)**	**(*****n*** **=** **14)**	**(*****N*** **=** **109)**	
Gender (% female)	13 (44.8%)	35 (53.0%)	8 (57.1%)	56 (51.4%)	0.257
Age (Mean, SD)	40.0 (11.9)	46.9 (13.0)	49.9 (12.3)	45.5 (13.0)	0.021
Married / defacto (%)	3 (10.3%)	9 (13.6%)	4 (28.6%)	16 (14.7%)	0.331
Not working (%)	22 (75.9%)	38 (57.6%)	4 (36%)	65 (59.6%)	0.014
Partially or fully supported accommodation (%)	19 (65.5%)	8 (12.1%)	2 (14.3%)	29 (26.6%)	<0.001
HSI (Mean, SD)	3.6 (1.5)	3.7 (1.2)	3.5 (1.4)	3.7 (1.3)	0.794
K10 (Mean, SD)	27.5 (7.4)	29.6 (7.8)	30.1 (8.2)	29.3 (7.8)	0.505
MINI diagnosis (% psychotic disorder)^#^	18/22 (82%)	34/58 (58.6%)	7/12 (58.3%)	59/92 (64.1%)	0.297
AUDIT C (% excessive alcohol consumption)	16 (55.2%)	27/65 (41.5%)	7 (50.0%)	50/108 (46.3%)	0.448
HRQL^*^ (Mean, SD)	0.567 (0.21)	0.503 (0.19)	0.502 (0.19)	0.520 (0.20)	0.352

# *Includes one person who received a diagnosis of borderline personality disorder on the McLean Screening Instrument for Borderline Personality Disorder*.

* *Health related quality of life (HRQL) utilities were elicited using AQOL-8D for face-to-face recruitments and the comparable EQ-5D plus four AQOL-8D bolt-on questions for postcard and online recruitments (35)*.

### Smoking

Participants began smoking regularly at a mean age of 16.7 years (SD 5.8). The mean number of cigarettes smoked per day at baseline assessment being 20.8 (SD 9.7), and the majority (*n* = 102; 93%) smoked their first cigarette within 30 min of waking. Most people smoked manufactured cigarettes (*n* = 83; 76%), around half smoked pouch tobacco (*n* = 54; 49.5%) and 27 (24.8%) smoked bulk tobacco. Over one-fifth (*n* = 25; 22.9%) smoked tobacco from butts that others left behind, with 9 of these doing so at least weekly.

### Psychosocial Functioning

Levels of psychological distress (K-10) were generally elevated, with 42/91 (46.2%) reporting very high, 31/91 (34.1%) high, 17/91 (18.7%) and moderate levels of distress. The mean score was 29.1 (SD 7.7). The MINI was delivered to 91 people; of whom four did not meet diagnostic criteria on any of the delivered modules (classified as “non-psychotic” for this analysis); as such 87 received a MINI diagnosis (see Table 1). Of the 18 not receiving the MINI, one had a self-reported primary diagnosis of borderline personality disorder and completed the McLean Screening Instrument for Borderline Personality Disorder (29). Only one person (recruited online) reported an alcohol problem without any co-existing mental health concern. Of those for whom MINI data were missing, eight did not complete any follow-up assessments (as such the MINI could not be administered); seven attended one assessment but the MINI was not administered due to time and/or rapport constraints; one requested to cease the questions; and one was unable to complete due to medical issues.

The sample mean HRQL utility score was 0.52, indicating relatively poor HRQL. For context, Engel et al. (37) recently assessed AQOL-8D utilities using data from Australia, Canada, Germany, Norway, UK and USA, and found a mean utilities score of 0.42 amongst adults with depression (*N* = 917) compared with 0.83 for healthy controls (*N* = 1,760). We found no significant difference in HRQOL utilities between recruitment strategies (face-to-face=0.57; postcard=0.50; online=0.50). It is important to note that lockdowns in response to COVID-19 and associated economic downturns may have impacted HRQL utilities amongst participants recruited via the postcard and online recruitment strategies.

### Profile of Study Participants According to Recruitment Method

Table 1 presents selected demographic, smoking and clinical characteristics of participants according to recruitment strategies. Reflecting our initial focus on recruiting from supported independent living accommodation facilities, participants recruited face-to-face were significantly more likely to be living in either partially or fully supported accommodation than those recruited online (OR = 11.40, 95% CI [2.12, 61.25], p = 0.003) or by postcard (OR = 13.78, 95% CI [4.75, 39.93], *p* = <0.001). As seen in Table 1, they also tended to be younger and more likely to be unemployed.

### Costing Analysis

The cost of training and equipping peer researchers was AU$27,253, and the total cost of recruiting, including peer researcher support and supervision was AU$128,878 at an average cost of AU$1,182 per participant recruited (Table 2). Personnel costs made up about 80% of recruitment phase costs, though only about 60% for online recruitment (see Supplementary Material Costing Tables for more detailed costing). Face-to-face recruitment was the most expensive at AU$1,648 per participant compared with the least expensive postcard recruitment costing an average AU$928 per participant recruited. However, face-to-face was relatively effective in reaching people currently living in supported accommodation, indicating that any trial that focused solely on such participants would be especially resource intensive in recruitment. Online recruitment was less resource intensive in terms of peer researcher and investigator time but yielded the fewest participants, though this was presumably at least partially a result of the online advertising budget constraints.

**Table 2:** Summary of study recruitment costs (AU$).

**Research project's cost breakdown**	**Training and equipping Peer Researchers**	**Recruiting costs**			
		**Face-to-Face**	**Postcard**	**Online**	**Total recruiting costs**
Research project personnel	$20,316	$41,768	$51,197	$11,987	$102,739
Equipment (incl. phone/tablet plans)	$4,411	$674	$240	$0^*^	$914
Travel (training, supervision & site visits)	$2,526	$3,007	$0	$0	$3,007
Advertising materials (paper-based)	$0	$1,139	$6,050	$0	$7,190
Advertising material (on-line, incl. website costs)	$0	$0	$0	$7,063	$7,063
Participant remuneration (vouchers + postage)	$0	$1,214	$3,768	$770	$5,752
**Total costs**	**$27,253**	**$47,803**	**$61,256**	**$19,820**	**$128,878**
Participants recruited	29	66	14	109
Average cost per participant recruited^**^		**$1,648.38**	**$928.12**	**$1,415.70**	**$1,182.37**

* *IT equipment for designing website etc. included in salary on-costs and advertising material costs as part of invoices*.

** *Excludes the costs of training and equipping peer researchers*.

## Discussion

Peer researcher facilitated recruitment into this tobacco treatment trial among people experiencing SMI was difficult and relatively expensive compared to recruitment to other smoking or mental health trials (16, 24). Even if all three recruitment strategies (face-to-face, direct mail postcards and online) had been able to occur simultaneously and assuming observed recruitment rates held constant over a longer recruitment period, it would have taken almost 3 years to achieve the target sample. Similarly, if we were able to identify and access new recipient population mail lists and just employed our most effective approach, direct mail postcards with telephone contact with peer researchers, without more peer researchers to field calls, it would take around 6 years. However, our *a priori* expectations were that recruitment of this socially marginalized population group to a smoking cessation study would require greater resources than other smoking and mental health studies (15), and important lessons have been learnt about peer researcher involvement, and the potential for direct mail postcards that may inform future research and clinical practice.

In the context of our RCT during the COVID-19 pandemic and with a limited timeline, recruitment was well below our original target. Unexpectedly, recruitment by peer researchers face-to-face before COVID-19 was slow, despite existing CI partnerships with participating mental health organizations. In contrast, the SCIMITAR+ Trial in the UK (38) recruited 526 participants into their tobacco treatment trial among people experiencing SMI (those with current drug or alcohol abuse were excluded) in just over a year. Successful recruitment was associated with use of NHS targets such as supporting access to research projects to encourage team engagement and establishing close working relationships between researchers and clinicians. Recruitment was via general practitioners, community mental health teams or psychiatrists, service user groups, poster advertisements and a lifestyle survey, all with suitability for participation established by a clinician. SCIMITAR researchers screened caseloads for eligible participants and attended the next meeting with the potential participant in order to discuss the study.

Shortly prior to our study commencing, there was a major shift in service delivery in Australia, with the introduction of Australia's National Disability Insurance Scheme (NDIS). This severely impacted our study. One of the participating mental health organizations decided not to engage with the study due to the restructuring process. The residential services which we did recruit from shifted focus from residential rehabilitation to supported independent living, with eligible residents' capacity to live independently being impacted by long-term mental ill-health and having levels of psychosocial disability that required assistance with activities for daily living. All residents of the supported accommodation facilities were eligible for NDIS support that requires evidence of permanent and significant disability that affects the individual's ability to take part in everyday activities. Thus, residents were people who may have faced significant challenges in participating in this trial without considerable support. This face-to-face recruitment was also relatively resource intensive. Prior to pivoting to postcard recruitment our average cost per enrollee via face-to-face was about $1,648 per participant. However, regardless of expense, different recruitment strategies and settings will provide different sub-samples, enhancing representativeness of the data.

It is possible that more frequent visits to residential settings by peer workers, working locally within teams, may have had better success. One report (39) of recruitment of people with schizophrenia into a coronary heart disease prevention intervention required an average 10.3 home occupational therapy visits to recruit a participant. The number of required visits were influenced by potential participants forgetting appointments, having difficulty assimilating study information, and also perceiving the generally welcomed research occupational therapist visits might terminate once consent was provided. Such frequent visits would not be possible in our study, with one of our participating organizations being widely dispersed throughout Victoria with multiple sites and different points of service delivery in urban areas and regional towns across long distances. Also, with the introduction of the NDIS and the shift to a more individualized funding approach, there was less group and center-based activities that would have enabled direct contact with potential participants.

We were unsuccessful in attracting mental health inpatients into our trial, although that had been successful in other studies (40). A potential explanation is that service delivery has become increasingly acute and pressured in recent years in Victoria, as detailed by the Royal Commission into Victoria's Mental Health Service System which was being conducted during the recruitment period (41), and together with COVID-19 restrictions, the inpatient setting was unlikely to become a source of recruitment during the study. Thus, although we had partnerships with the recruiting organizations, our peer researchers were not as strongly embedded within services as they were in the SCIMITAR+trial (39) and we were unable to make as many visits to sites as others have done due to statewide and dispersed service delivery (39). Peer researchers were facing services that had competing priorities and significant organizational disruption.

In future, establishing organizational targets for delivery of smoking interventions and possibly pairing peer workers with clinician champions in further studies or in clinical contexts may help build closer working relationships and better uptake of interventions in residential and inpatient contexts. Although challenging, it is important people within these settings receive opportunities for tobacco treatment and for representativeness in research. It is possible that financial incentives for service providers to refer could be effective because of the time constraints and overwhelming administrative work that compete with research and represent important barriers (42). In addition, although peer researchers may be able to connect effectively with consumers, there appears to be a need for alternative strategies and different messaging to engage staff and managers and explain the benefits of a trial such as this. RCTs can be an even harder “sell” when staff know only half of participants will receive an active intervention. In the current trial, we limited disclosure about intervention arm contents, but staff sometimes asked whether participants would all receive NRT. Future research needs to align with service organization goals and strategy at all levels—executive, managerial, and for practitioners. In the case of the present study, service organization goals and strategies may have been shifting quickly with the advent of the NDIS. With so many pressures, organizations are likely to prioritize activities with a clear pay off for clients and the organization.

As recruitment from community residential facilities was comparatively low and unsuccessful from acute inpatient units in this study, additional ways to link mental health consumers in those settings to quitline is worthy of further investigation. For example, in a randomized trial with 224 individuals recruited from a locked acute psychiatry unit with a smoking ban, verified smoking 7-day point prevalence abstinence over 18-months follow-up was significantly higher for those who received a computer-assisted tobacco intervention with posthospitalization NRT (20.0%) vs. usual care (7.7%) (43). Such computer delivered interventions could also provide referrals to quitline, with quitline staff potentially beginning communication with people who smoke via text or telephone, with follow-up after discharge.

Recruitment via direct mail postcards, inviting people registered with mental health organizations who smoked to telephone peer researchers to find out more about the study, was relatively successful. We began sending out postcards a year after face-to-face recruitment commenced, hoping to improve the rate of recruitment through reaching more people and to recruit in a COVID-safe manner. As described above, peer researchers obtained verbal consent via telephone from participants and a shorter baseline assessment was implemented. People recruited via postcard did not differ from other recruitment strategies, apart from residential status. Our recruitment rate (1.6%; 66 participants from postcards to approximately 4200 people over two occasions) is similar to that of other health intervention studies recruiting by postcard. For example, in a study recruiting young people for a randomized trial of weight gain prevention interventions, Crane et al. (44) mailed postcards once to 30,000 people, with 30 being randomized into the study (1.3% response rate), costing US $7,422; $247.40 per participant. They found little difference in reach between postcards and brochures (sent to a separate sample). Waltman et al. (45) mailed postcards to 72,469 women and resent them 6 months later, with 47 participants enrolling in a RCT of different interventions on bone health (0.07% response rate). The total cost of postcard recruitment was US $43,567.49; $926.96 per participant (the cost of researchers' time in implementing recruitment strategies was not considered in the calculation), which is comparable to our average cost of postcard recruitment of AU$928 per participant (including researcher time), suggesting recruitment to trials requiring difficult behavior change can be expensive. In Waltman et al. (45), postcards were the second most successful recruitment strategy after health care provider letters (*n* = 58) and similar to Facebook posts (*n* = 44); lower numbers were obtained from referral by family and friends (*n* = 11), newspaper or television advertisements (*n* = 5) and digital advertisements (*n* = 2). Waltman et al. regarded health care provider letters and postcards as successful in helping to reach their overall target of 275 participants. In hindsight, we could have had postcard recruitment running alongside face-to-face recruitment and may have been able to attract about 66 people annually, this would likely have introduced some efficiencies in peer researcher and investigator supervision time. In the present study, the mental health organizations involved did not have a record of smoking status so we had to send postcards to all people registered by the organizations. It would obviously be cheaper to send postcards if registries kept smoking status and other risk factor information for targeted health marketing. However, sending postcards to everyone has the advantage of capturing new and unrecorded smokers. Response to postcards was stronger following the second mail out. Peer researchers reported that respondents often commented that the second postcard prompted them to call. Even though we did not reach our recruitment target for the study, direct mail postcards every 6 months to people registered with mental health services, with the option of phoning a peer worker or quitline directly, may be a relatively effective way of increasing contact with quitlines.

Our online recruitment strategy was the least costly in total (AU$19,820) but was only implemented for the 4 months before trial closure, resulting in 14 participants ($1,416 per participant). Qualitative research with participants is currently underway to develop a more in-depth understanding of participant experiences. However, we do not have any information about why several thousand views online led to so few enrolments. It is possible the lengthy information and consent forms mandated by ethics committees did not engage people sufficiently online. Interestingly, a recent study of recruitment into an online intervention among people with SMI aimed for a sample size of 148 over 2 years and recruited only 98 (46). Had we started online recruitment at the commencement of our study, and assuming a similar rate over our 2-year recruitment period, we would have recruited about 84 participants by that method.

There is a paucity of directly comparable cost analyses for recruitment to smoking cessation studies. Buller et al. (24) recruited participants at a much lower cost per participant, but they did not target people who experience SMI. Whilst hindsight reveals some potential sources of efficiencies for our project, recruiting to such studies is resource intensive. However, it is important to put this in the context of the potential cost offsets to be gained from facilitating the prevention of smoking related illness. For example, Golsbury et al. estimate the average additional health costs for an Australian diagnosed with lung cancer between 45 and 60 years old is AU$67,689 (47). In addition, a qualitative study among the participants of the present trial reported that the peer researcher and quitline interventions in the Quitlink study have been highly valued as compassionate approaches that have the potential to assist people on a journey to quitting (McCarter et al., submitted^1^).

Training of peer researchers for this study, preparation of a detailed peer researcher manual and ongoing supervision was necessary as the peer researchers had not assisted on an RCT before and the baseline assessments were initially quite long and were administered on an iPad linked to REDcap. One solution may have been to have experienced research assistants work alongside the peer researchers. However, once such peer researchers are trained, they form an important element of the peer researcher workforce to be engaged with future studies and also help to train and support others. In Australia, the peer research workforce is limited and needs support and resourcing for future development. Lived experience is increasingly seen as a discipline, with potential of forming a recognized profession (48). This also enhances the potential for co-design of strategies to improve research activities, including recruitment. Alongside the need for this level of peer researcher training and expertise, efficiencies in postcard recruitment, involving telephone contact with peer researchers and a briefer assessment conducted over the phone (in the context of a RCT), could be a useful model for peer telehealth interventions in the age of COVID-19 but also more broadly with widely dispersed services. Nevertheless, structural impediments to postcard effectiveness, such as unstable housing and people not receiving postcards mean that face-to-face advice regarding tobacco treatment should remain an important staple of usual care.

### Peer Researcher Commentary

#### The Process of Research

Generally speaking, past research has been critiqued for “othering” mental health participants. This means that it may omit or misunderstand details which are important to the people whom we are trying to support. This might also impact the degree to which research can define or address the problem. Future studies are encouraged to utilize co-design and co-production, which is the practice of including people with lived experience in the design and conduct of the study. This is potentially a challenging process for researchers as it subverts the traditional power dynamic but also allows for new opportunities to connect with participants, which may lead to better recruitment and richer data.

#### Conducting the Research

Many randomized controlled trials are designed to elicit as much information from participants as possible but may not take into account how this impacts the participant (e.g., difficulty due to literacy, length of surveys, intrusive questions and their mental health). For example, around half the participants in this study had not completed further education after high school; this is when peer researchers can support participants to feel that the project is accessible and that they understand their rights, including their right to not participate or withdraw.

#### The Challenges With Mental Health Services

Peer researchers were responsible for managing multiple complex referral pathways across the state and between partner organizations. At times, the peer researchers felt that recruitment was impacted by poor staff engagement and gatekeeping. This, however, was not an issue during the postcards recruitment as they were delivered directly to the potential participants who were able to choose if and when to contact the peer researcher who was supporting recruitment. Overall, the peer researchers felt like valued members of the research team and helped the team to understand future opportunities for consumer involvement through working and learning together.

#### Understanding the Experience of Quitting Smoking

Many consumers consider smoking to be an important part of their lives. Smoking has connotations of social exclusion which can further marginalize the participants of this study. This study is important because the baseline assessment questions inquired into sources of smoking that are deeply stigmatized, such as discarded cigarette butts. In this way, it is understood that smoking is broader than just a health issue and it has deep social and economic consequences.

#### The Shared Experience of Quitting Smoking

Importantly, some of the peer researchers also had the lived experience of being a smoker and quitting smoking. Having experienced many common challenges (e.g., peer pressure to keep smoking, boredom, difficulty accessing or using NRT) often increased mutuality and connection between the peer researchers and the participants. The peer researchers felt that their experience, or knowledge of other people's quitting journey, helped lessen stigma and created a non-judgmental and understanding space to explore the topic. The importance of access to NRT was highlighted in these discussions.

#### Support for Peer Researchers

Also, the personal impact of hearing people's personal stories was acknowledged by the peer researchers as discussion about quitting also involved sharing heart felt or challenging experiences. Peer researchers found that setting a limit of two interviews a day was manageable in terms of self-care and administrative burden. Upon reflection, the peer researchers recommend developing debriefing, more opportunities for peer-to-peer support and access to peer supervision from a more experienced peer researcher, and other potential support for the peer researchers as a part of the study design for future studies. Future studies are encouraged to include lived experience investigators, this can help anchor the lived experience perspective and increase the ease with which the study can provide support to its peer workforce.

## Limitations

One of the main limitations of the present study is that we failed to recruit the intended sample of 382 people. However, this experience allowed us to adapt our recruitment strategies, and compare them. Nevertheless, it should be noted that the three strategies did not occur concurrently and were active for varying lengths of time. Further, the proportion of people included in the trial via each recruitment strategy may not be truly representative of all those invited or offered recruitment in the trial. Another limitation is that we did not compare peer researcher recruitment with alternative non-peer strategies of recruitment. Alternative approaches, such as computer delivered information about the study, accompanied by a brief intervention with a link to quitline in residential settings or use of “opt out” rather than “opt in” strategies when people who smoke newly present to mental health services may have yielded different results. There remains an opportunity to co-design these strategies with people with lived experience including peer researchers. COVID-19 may have confounded some of the measures in the study, with smoking and other substance use potentially rising and quality of life declining during the pandemic.

Changes in investigators may have influenced recruitment, with CI Brophy departing one of the main organizations from which we recruited early in the study and CI Castle leaving the other main site later in the study, potentially lessening active commitment from organizations involved. Our peer researcher lead, who had been very active in developing the study, retired just as the study began. In hindsight, a replacement for a peer researcher lead may have addressed peer researcher needs for supervision in addition to that provided by the CI.

The main organizations in the study were comprised of widely dispersed services, necessitating travel over long distances. As peer researchers were embedded at head offices of the organizations, developing an ongoing recruitment routine was difficult. Future studies may more fruitfully employ peer researchers already attached to local services to establish recruitment protocols into practice. Victoria's clinical services have become more oriented to crisis care and are characterized by supporting large numbers of people considered to have SMI with complex needs and many are on compulsory orders (41). This challenging service delivery environment may have lowered expectations of staff and contributed to lower recruitment.

In terms of methodology, we did not audio record peer researchers' interactions with participants to monitor fidelity to the recruitment procedures. Peer researchers thought recording interactions may have been declined by most people. However, some measure of fidelity, perhaps a checklist, may have given a better sense of fidelity to the peer recruitment manual. On the other hand, at commencement on the project, CIs shadowed peer researchers in delivering the recruitment information and recruitment process, observing baseline assessment, randomization and feedback to participants. Finally, some participants did not complete the MINI, so diagnosis is only available on 91 people.

## Conclusions

Recruitment of a broad range of people experiencing SMI (i.e., including those with alcohol and other drug issues and those living in supported accommodation) into our smoking intervention study was difficult and expensive. The recruitment rate we achieved was far lower than targeted and required us to adapt and develop a range of recruitment strategies. Face-to-face, direct mail postcard followed by telephone contact and online recruitment required different degrees of peer researcher involvement. These recruitment strategies could run in parallel to help attract people experiencing SMI into smoking interventions in clinical settings. This study relied on the commitment of partner organizations that are often operating in the context of competing priorities. Maintaining engagement from when a research project is formulated through to its implementation requires consistent and thoughtful planning that considers changes in leadership and other disruptions. Acknowledging that staff have an important role to play in enabling recruitment requires ensuring they are supported to understand the value of tobacco treatment. A proactive longer-term view of continually recruiting into tobacco treatment is needed in community mental health organizations, alongside preventive approaches to discourage uptake of smoking. Early and continued physical health intervention from first mental health presentation is vital (5) and tobacco treatment should be part of this approach.

## Data Availability Statement

Data is available upon request subject to approval.

## Ethics Statement

The studies involving human participants were reviewed and approved by St Vincent's Hospital Melbourne Human Research Ethics Committee (HREC Reference Number: HREC/18/SVHM/154), the University of Newcastle Human Research Ethics Committee (HREC Reference Number: H-2018-0192) and the Cancer Council Victoria Human Research Ethics Committee (HREC Reference Number: 1807). The patients/participants provided their written, verbal (audiotaped), or digital consent to participate in this study.

## Author Contributions

The first draft of the paper was written by ALB with significant input from KM, RS, LB, NC, CB, and MLM followed by the remaining authors. Statistical analyses were conducted by JA, DL, and DEB. All authors contributed to the design and write up of the study.

## Funding

This project was funded by the National Health and Medical Research Council (APP1139125). The funder had no role in study design, data collection and analysis, decision to publish, or preparation of the manuscript. ALB holds a NHMRC Fellowship (APP1135901) and Faculty of Health and Medicine, University of Newcastle, Gladys M. Brawn Fellowship. KM holds a University of Newcastle Postdoctoral Scholarship. DEB was funded by a University of Newcastle Ph.D. scholarship.

## Conflict of Interest

NC and LH were employed by MIND Australia. MIND Australia is a not for profit, non-government organisation that receives public funding and private donations.

## Publisher's Note

All claims expressed in this article are solely those of the authors and do not necessarily represent those of their affiliated organizations, or those of the publisher, the editors and the reviewers. Any product that may be evaluated in this article, or claim that may be made by its manufacturer, is not guaranteed or endorsed by the publisher.

## Footnotes

^1^McCarter K, McKinlay M, Cocks N, Brasier C, Hayes L, Baker A, et al. (submitted). The Value of Compassionate Support to Address Smoking: A Qualitative Study With PeopleWho Experience Severe Mental Illness.
