# Supplementary material for: Adapting Peer Researcher Facilitated Strategies to Recruit People Receiving Mental Health Services to a Tobacco Treatment Trial
Source: Front Psychiatry. 2022 May 26;13:869169. doi: 10.3389/fpsyt.2022.869169 (PMC9199858; doi:10.3389/fpsyt.2022.869169)
Supplement: Supplementary file 1 [file Data_Sheet_1.ZIP › Revised Supplementary Material/Supplementary Material - Detailed Costing by Recruitment Phase (AU$)_070422.docx]

| **i. Peer Researcher Training & Equipment (2018)** | | | | | | | | | |
| --- | --- | --- | --- | --- | --- | --- | --- | --- | --- |
| **Resource** | **Activity** | **Units** | **No. units** | **Cost/unit*** | **Cost/unit 2021 AU$**** | **Total cost** | **Assumptions/rates/notes** | **Source of quantity data** | **Source of unit cost data** |
| Peer Researcher (PR) time | (i)Developing Peer Researcher Operations Manual; (ii) Attending training (58 hrs of training sessions provided by Prof, Assoc.Prof & RFs, 11 sessions for 1 or 2 PRs) | days | 12 | $454.09 | $454.09 | $5,449.13 | 2021 salary for HEW-6, Step 2 + 38%on-costs | (i) Investigator (KM) recall; (ii) Project records | University of Newcastle 2021 salary rates |
| Investigator time (Prof.) | Deliver Peer Researcher training sessions | hours | 30.5 | $148.71 | $148.71 | $4,535.64 | 2021 salary for Level E + 38% on-costs | Project records | University of Newcastle 2021 salary rates |
| Investigator time (Assoc. Prof) | Deliver Peer Researcher training sessions | hours | 21 | $119.35 | $119.35 | $2,506.44 | 2021 salary for Level D, Step 2 + 38% on-costs | Project records | University of Newcastle 2021 salary rates |
| Investigator time (Research Fellow level B) | (i) Developing Peer Researcher Operations Manual; (ii) Deliver Peer Researcher training sessions | days | 13.3 | $588.95 | $588.95 | $7,824.58 | 2021 salary for Level B, Step 3 + 38%on-costs | Investigator (KM) recall | University of Newcastle 2021 salary rates |
| Venue hire for training | Used offices of investigators - utilities and space covered in salary on-costs |  |  |  |  | $0.00 | We used offices of investigators - the cost of these spaces are captured in salary on-costs |  |  |
| Travel: Rtn flights, 1 night accomm, transfers & meals | Prof & Assoc.Prof. travel Newcastle to Melbourne to train Peer Researchers (2019). Peer Researcher travel Melbourne to Newcastle for training (2019). | travel package units | 3 | $804.64 | $842.08 | $2,526.24 | Rtn flights, 1 night accomm, transfers & meals. Expenses inflated from 2019 (2.3% annual inflation). | Project records | Project invoices |
| Ipad pro 12.9 (incl keyboard, screen protectors + pencils + cases) | Used for screening potential participants and also assessment | ipad pack | 2 | $1,832.41 | $1,948.38 | $3,896.77 | Ipad pro 12.9 (incl keyboard and accessories). Expenses inflated from 2018 expense (2.1% annual inflation). | Project records | Project invoices |
| Ipad monthly plan | Screening and baseline data collection | monthly plans | 1 | $10.00 | $10.00 | $10.00 | No inflation as monthly plan costs have not changed | Project records | Project invoices |
| Mobile phone handsets | Required for supervision calls and subsequent phases of participant recruitment | phones | 2 | $247.00 | $247.00 | $494.00 | Smartphone 2021 prices | Project records | Project invoices |
| Mobile monthly plan | Required for supervision calls and subsequent phases of participant recruitment | monthly plans | 1 | $10.00 | $10.00 | $10.00 | No inflation as monthly plan costs have not changed | Project records | Project invoices |
|  |  |  |  |  | **TOTAL** | **$27,253** |  |  |  |

*Universities are generally exempt from Australian goods and services tax (10%). Costs incurring GST, are flagged. ** Annual inflation from Reserve Bank of Australia: <https://www.rba.gov.au>.

| **ii. Face to face recruitment (March 2019 to April 2020)** | | | | | | | | | |
| --- | --- | --- | --- | --- | --- | --- | --- | --- | --- |
| **Resource** | **Activity** | **units** | **no. units** | **cost/unit*** | **cost/unit 2021 AU$**** | **total cost** | **Assumptions/Rates/Notes** | **Source of quantity data** | **Source of unit cost data** |
| Peer Researcher time | Recruiting activities (calling and visiting sites, calls with potential participants, conducting screening & baseline surveys, attending fortnightly supervision calls & admin) | days | 74 | $454.09 | $454.09 | $33,792.18 | 2021 salary for HEW-6, Step 2 + 38%on-costs; assumes PR spent half of their 2days/wk directly on recruitment activities (74 PR days = 4 days/mth x 18.5 PR months) | Project records & Investigator (AB, KM) recall | University of Newcastle 2021 salary rates |
| Investigator time (Prof.) | Peer Researcher fortnightly supervision calls + visit | hours | 18.8 | $148.71 | $148.71 | $2,795.74 | 2021 salary for Level E + 38% on-costs | Project records | University of Newcastle 2021 salary rates (see "salaries" worksheet for link) |
| Investigator time (Assoc. Prof.) | Peer Researcher fortnightly supervision calls + visit | hours | 13.25 | $119.35 | $119.35 | $1,581.44 | 2021 salary for Level D, Step 2 + 38% on-costs | Project records | University of Newcastle 2021 salary rates |
| Investigator time (Research Fellow  level B) | Peer Researcher fortnightly supervision calls | hours | 23.21 | $84.14 | $84.14 | $1,952.78 | 2021 salary for Level B, Step 3 + 38%on-costs | Project records | University of Newcastle 2021 salary rates |
| Investigator time (Research Fellow  level A) | Designed & managed Peer Researcher potential participant call tracking sheet | hours | 11.5 | $64.18 | $64.18 | $738.06 | 2021 salary for Level A, Step 3 + 38%on-costs | Investigator (AB, CB) recall | University of Newcastle 2021 salary rates |
| Project Administrator/Research Assistant time | Designing posters, flyers and postcards for display at sites | days | 2 | $454.09 | $454.09 | $908.19 | 2021 salary for HEW-6, Step 2 + 38%on-costs | Project records | Project invoices |
| Travel: Flights + accomm + taxi package + meals | Prof & Assoc. Prof. travel for in-person supervision & training | Travel packages | 2 | $804.64 | $842.08 | $1,684.16 | Investigator Trip includes: flights, 1 night accomm, transfers & meals. Expenses inflated from 2019 (2.3% annual inflation). | Project records | Project invoices |
| Travel: Peer Researcher vehicle for day site visits | Driving around greater Melbourne for Site Visits | car costs | 1 | $299.30 | $313.23 | $313.23 | Peer Researcher personal car costs = $0.72 per km driven (petrol, maintenance, depreciation). Expenses inflated from 2019 (2.3% annual inflation). | Project records | <https://www.ato.gov.au/Business/Income-and-deductions-for-business/Deductions/Deductions-for-motor-vehicle-expenses/Cents-per-kilometre-method/> |
| Travel: Peer Researchers accomm, travel and meals for overnight site visit | Wodonga site visit | travel package Wodonga | 2 | $482.56 | $505.01 | $1,010.03 | Train ticket, 1 night accomm & meals. Expenses inflated from 2019 (2.3% annual inflation). | Project records | Project invoices |
| Printing posters, flyers & postcards | Printing posters, flyers & postcards for display at MH Sites | print run | 1 | $1,088.60 | $1,139.25 | $1,139.25 | Expenses inflated from 2019 (2.3% annual inflation). | Project records | Project invoices |
| Cash box | Storing gift card vouchers during site visits | cash box | 2 | $31.40 | $32.86 | $65.72 | Expenses inflated from 2019 (2.3% annual inflation). | Project records | Project invoices |
| Trolley bag | Required for equipment, flyers, cash box, etc., on site visits | trolley bag | 2 | $109.00 | $114.07 | $228.14 | Expenses inflated from 2019 (2.3% annual inflation). | Project records | Project invoices |
| Mobile monthly plan | Communicating with Investigators, participating mental health services and participants | monthly plans | 19 | $10.00 | $10.00 | $190.00 | No inflation as monthly plan costs have not changed | Project records | Project invoices |
| Ipad monthly plan | Screening and baseline data collection | monthly plans | 19 | $10.00 | $10.00 | $190.00 | No inflation as monthly plan costs have not changed | Project records | Project invoices |
| Gift cards vouchers | Gift car voucher to thank participants | vouchers | 29 | $40.00 | $41.86 | $1,213.97 | Expenses inflated from 2019 (2.3% annual inflation). | Project records | Project invoices |
| Gift card voucher express bag postage | Postage of gift card vouchers to participants | express post bags | 0 |  |  |  | Gift cards handed out in person for face to face recruits | Project records | Project invoices |
|  |  |  |  |  | **TOTAL** | **$47,802.90** |  |  |  |

*Universities are generally exempt from Australian goods and services tax (10%). Costs incurring GST, are flagged. ** Annual inflation from Reserve Bank of Australia: <https://www.rba.gov.au>.

| **iii. Postcard recruitment (April 2020 to April 2021)** | | | | | | | | | |
| --- | --- | --- | --- | --- | --- | --- | --- | --- | --- |
| **Resource** | **Activity** | **units** | **no. units** | **cost/unit*** | **cost/unit 2021 AU$**** | **total cost** | **Assumptions/Rates/Notes** | **Source of quantity data** | **Source of unit cost data** |
| Peer Researcher time | Recruiting activities (liaising with MH Orgs/sites, calls with potential participants, conducting screening & baseline surveys, attending fortnightly supervision calls & admin) | days | 93 | $454.09 | $454.09 | $42,230.77 | 2021 salary for HEW-6, Step 2 + 38%on-costs; assumes PR spent half of their 2days/wk directly on recruitment activities (=2 x PRs @ 46.5 days each = 93 days) | Project records & Investigator (AB, KM) recall | University of Newcastle 2021 salary rates |
| Investigator time (Prof.) | Peer Researcher fortnghtly supervision calls | hours | 21.1 | $148.71 | $148.71 | $3,136.28 | 2021 salary for Level E + 38% on-costs | Project records | University of Newcastle 2021 salary rates |
| Investigator time (Assoc. Prof.) | Peer Researcher fortnghtly supervision calls | hours | 2.9 | $119.35 | $119.35 | $348.51 | 2021 salary for Level D, Step 2 + 38% on-costs | Project records | University of Newcastle 2021 salary rates |
| Investigator time (Research Fellow  level B) | i. Peer Researcher supervision calls; ii.design post cards (2 days) | hours | 27.8 | $84.14 | $84.14 | $2,334.75 | 2021 salary for Level B, Step 3 + 38%on-costs | Project records | University of Newcastle 2021 salary rates |
| Investigator time (Research Fellow  level A) | Designed & managed Peer Researcher potential participant call tracking sheet | hours | 23 | $64.18 | $64.18 | $1,476.12 | 2021 salary for Level A, Step 3 + 38%on-costs | Investigator (AB, CB) recall | University of Newcastle 2021 salary rates |
| Project Administrator/Research Assistant time | Admin in managing postcard and gift card voucher mail-outs | days | 2 | $835.48 | $835.48 | $1,670.96 | 2021 Salary for HEW-6, Step 2 + 38%on-costs | Project Admin officer recall | University of Newcastle 2021 salary rates |
| Printing post cards | printing cards | batch | 1 | $957.92 | $994.32 | $994.32 | Inflation applied (3.8% annual inflation) | Project records | Project invoices |
| Posting post cards | Post costs | batch | 1 | $4,870.90 | $5,055.99 | $5,055.99 | Inflation applied (3.8% annual inflation) | Project records | Project invoices |
| Mobile monthly plan | Communicating with Investigators, participating mental health services and participants | monthly plans | 12 | $10.00 | $10.00 | $120.00 | No inflation as monthly plan costs have not changed | Project records | Project invoices |
| Ipad monthly plan | Screening and baseline data collection | monthly plans | 12 | $10.00 | $10.00 | $120.00 | No inflation as monthly plan costs have not changed | Project records | Project invoices |
| Gift cards vouchers | Gift car voucher to thank participants | vouchers | 66 | $40.00 | $41.52 | $2,740.32 | Inflation applied (3.8% annual inflation) | Project records | Project invoices |
| Gift card voucher express bag postage | Postage of gift card vouchers to participants | express post bags | 66 | $15.00 | $15.57 | $1,027.62 | Inflation applied (3.8% annual inflation) | Project records | Project invoices |
|  |  |  |  |  | **TOTAL** | **$61,255.65** |  |  |  |

*Universities are generally exempt from Australian goods and services tax (10%). Costs incurring GST, are flagged. ** Annual inflation from Reserve Bank of Australia: <https://www.rba.gov.au>.

| **iv. Online recruitment (December 2020 to April 2021)** | | | | | | | | | |
| --- | --- | --- | --- | --- | --- | --- | --- | --- | --- |
| **Resource** | **Activity** | **units** | **no. units** | **cost/unit*** | **cost/unit 2021 AU$**** | **total cost** | **Assumptions/Rates/Notes** | **Source of quantity data** | **Source of unit cost data** |
| Peer Researcher time | Writing and recording recruitment videos for website | days | 0.5 | $454.09 | $454.09 | $227.05 | 2021 salary for HEW-6, Step 2 + 38%on-costs | Investigator (KM) recall | University of Newcastle 2021 salary rates |
| Investigator time (Assoc. Prof.) | Writing, recording (2days) & editing (0.5 days) recruitment videos for website | days | 2.5 | $835.48 | $835.48 | $2,088.70 | 2021 salary for Level D, Step 2 + 38% on-costs | Investigator (PK) recall | University of Newcastle 2021 salary rates |
| Investigator time (Research Fellow  level B) | Writing and recording recruitment videos for website (0.5day); Writing advertisement text and photos for Facebook ads (0.5 day) | days | 1.0 | $588.95 | $588.95 | $588.95 | 2021 salary for Level B, Step 3 + 38%on-costs | Investigator (KM) recall | University of Newcastle 2021 salary rates |
| Project Administrator time | Designing website content | days | 15 | $514.67 | $514.67 | $7,719.99 | 2021 salary for HEW 7, Step 3 + 38% on-costs | Project records | University of Newcastle 2021 salary rates |
| Project Administrator (2) time | Setting up Facebook ads; Admin in managing postcard and gift card voucher mail-outs (1 day) | days | 3 | $454.09 | $454.09 | $1,362.28 | 2021 salary for HEW-6, Step 2 + 38%on-costs | Project Admin officer recall | University of Newcastle 2021 salary rates |
| Online ads | Facebook costs | package | 1 | $5,122.93 | $5,122.93 | $5,122.93 | 3 ads over 6 months. Some broad and semi-target to "smoking"; note Facebook charged GST. | Project records | Project invoices |
| Website | Website Structure Design & hosting |  | 1 | $1,900.00 | $1,900.00 | $1,900.00 | External designer | Project records | Project invoices |
| Website | Domain name |  | 1 | $39.98 | $39.98 | $39.98 | No inflation applied to 2021 expenses | Project records | Project invoices |
| Mobile monthly plan | Communicating with Investigators, participating mental health services and participants | monthly plans | 0 | $10.00 | $10.00 | $0.00 | No inflation applied to 2021 expenses | Project records | Project invoices |
| Ipad monthly plan | Screening and baseline data collection | monthly plans | 0 | $10.00 | $10.00 | $0.00 | No inflation applied to 2021 expenses | Project records | Project invoices |
| Gift cards vouchers | Gift car voucher to thank participants | vouchers | 14 | $40.00 | $40.00 | $560.00 | No inflation applied to 2021 expenses | Project records | Project invoices |
| Gift card voucher express bag postage | Postage of gift card vouchers to participants | express post bags | 14 | $15.00 | $15.00 | $210.00 | No inflation applied to 2021 expenses | Project records | Project invoices |
|  |  |  |  |  | **TOTAL** | **$19,819.87** |  |  |  |

*Universities are generally exempt from Australian goods and services tax (10%). Costs incurring GST, are flagged. ** Annual inflation from Reserve Bank of Australia: <https://www.rba.gov.au>.
