# Supplementary material for: Adapting Peer Researcher Facilitated Strategies to Recruit People Receiving Mental Health Services to a Tobacco Treatment Trial
Source: Front Psychiatry. 2022 May 26;13:869169. doi: 10.3389/fpsyt.2022.869169 (PMC9199858; doi:10.3389/fpsyt.2022.869169)
Supplement: Supplementary file 1 [file Data_Sheet_1.ZIP › Revised Supplementary Material/Supplementary material - QuitLink social media posts.docx]

**Supplementary Material - QuitLink Study Social Media**

**Caption bank**

***Caption 1:*** Are you interested in getting control of your smoking or stopping completley?

Our researchers are currently looking to better understand how to help people who are accessing treatment and support for mental health, drug and alcohol conditions to stop smoking.

If you are aged 18+, currently smoke 10 or more ciggarettes a day, are accessing tretament or support for mental health, drug or alcohol condition, visit (link) to expres your interest in receiving assistance and information that can help you.

***Caption 2:*** Become smoke-free and live better!

Our researchers are looking at better understanding how to help people become smoke-free and live a healthier life. Can you help?

***Caption 3:*** Become smoke-free and live better!

Our researchers are seeking volunteers to participate in Quitlink – a study which will help us better understand ways to help people with mental health, drug and alcohol conditions reduce or stop smoking. Join QuitLink at (link).

***Caption 4:*** Are you interested in getting control of your smoking or stopping completley?

Our researchers are looking at better understanding how to help people become smoke-free and live a healthier life. Can you help? Participate in QuitLink today.

***Caption 5:*** If your interested in hearing more about how to get in control of your smoking, we’re interested in hearing from you.

Our researchers are currently looking to better understand how to help people who are accessing treatment and support for mental health, drug or alcohol conditions to stop smoking.

Express your interest in QuitLink today (link)

***Caption 6:*** Are you interested in getting control of your smoking or stopping completley/? If you are interested in receiving some information to help you reduce or stop smoking, express your interest in the QuitLink study.

***Caption 7:*** If you are ged 18+, currently smoke 10+ ciggarettes a day, are accessing trteament or support for mental health, drug or alcohol conditions, visit QuitLInk to express your interest in receiving assistance and information that can help you.

***Caption 8:*** Quitting smoking can often be difficult, especially for people receiving treatment for a mental health, drug or alcohol conditions. Find out how you can help!

**Facebook Sponsored post bank**

| 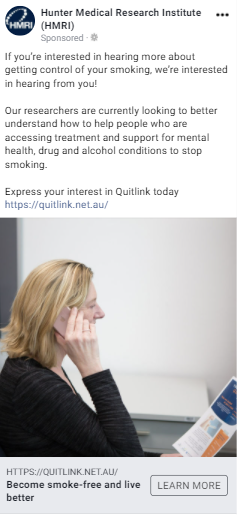 | 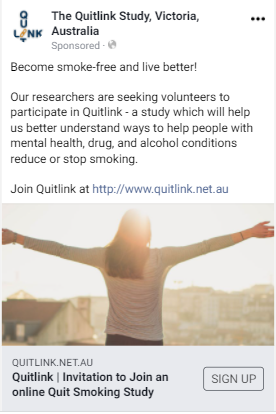 |
| --- | --- |
| 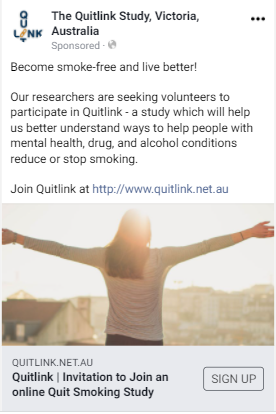 | 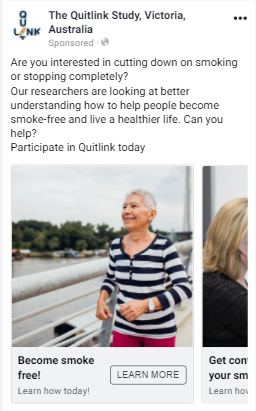 |
| 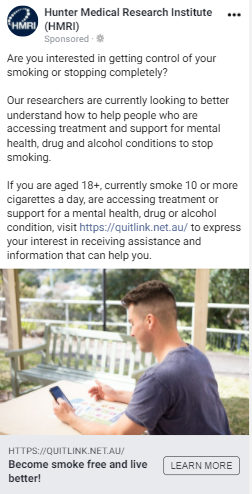 | 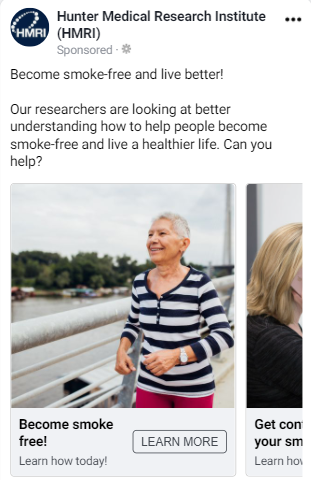 |
| 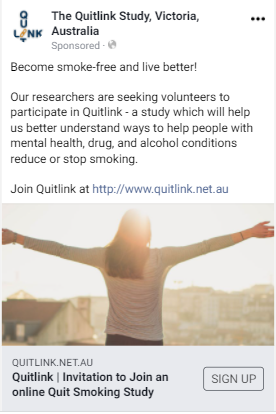 | 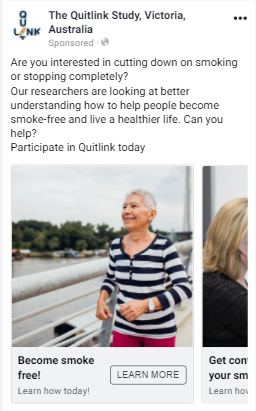 |
