# Supplementary material for: Adapting Peer Researcher Facilitated Strategies to Recruit People Receiving Mental Health Services to a Tobacco Treatment Trial
Source: Front Psychiatry. 2022 May 26;13:869169. doi: 10.3389/fpsyt.2022.869169 (PMC9199858; doi:10.3389/fpsyt.2022.869169)
Supplement: Supplementary file 1 [file Data_Sheet_1.ZIP › Revised Supplementary Material/Supplementary material - Quitlink postcards.pdf]

The QuitLink Study

QUIT LINK

You are invited to join a study

led by researchers at the University of Newcastle in conjunction with Cancer Council Victoria, Mind and St Vincent's Hospital Victoria.

If you are receiving **mental health services** and **smoke at least 10 cigarettes per day**, this study may interest you.

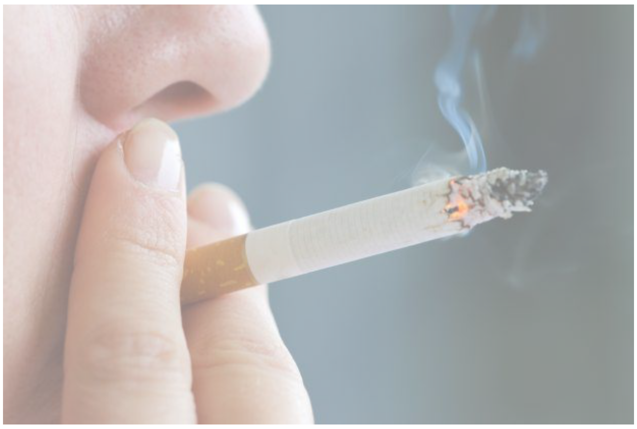

If you are aged **18 years or older** and are interested in finding out about the study or be considered for participation, please contact:

Peer worker
Ph: XXXX
Email: quitlink@newcastle.edu.au

Involvement in this study will require your commitment to:

- complete a touchscreen survey
- three telephone surveys
  - 2 months
  - 5 months
  - 8 months later

DEAKIN UNIVERSITY · mind Help, hope and purpose · MONASH University · UNIVERSITY OF WOLLONGONG AUSTRALIA · Cancer Council Australia · THE UNIVERSITY OF MELBOURNE · ST VINCENT'S HOSPITAL MELBOURNE · THE UNIVERSITY OF NEWCASTLE AUSTRALIA

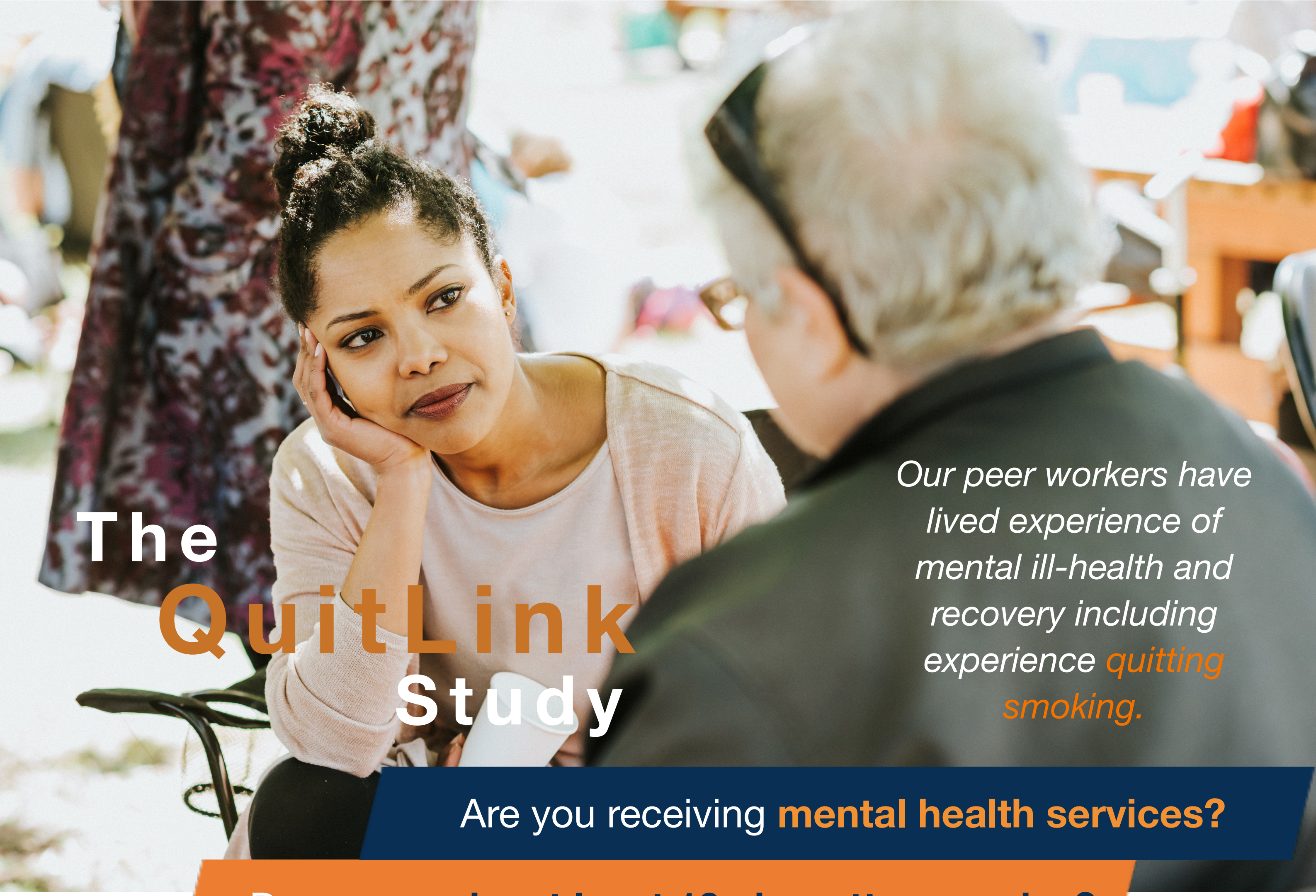

The QuitLink Study

Our peer workers have lived experience of mental ill-health and recovery including experience quitting smoking.

Are you receiving mental health services?

Do you smoke at least 10 cigarettes per day?

You are invited to join our study.

If you agree to take part you will be offered some help to stop smoking.

You will be asked to complete four telephone surveys (over 8 months).

You will receive a $40 gift card for each completed survey.

For more information, please call or text one of our peer workers:

Mon & Wed
XXXX

Tues & Thurs
XXXX

Fri
XXXX

OR email:
quitlink@newcastle.edu.au

Deakin University · mind (Help, hope and purpose) · Monash University · University of Wollongong Australia · Cancer Council Australia · The University of Melbourne · St Vincent's Hospital Melbourne · The University of Newcastle Australia
